# *Rickettsia typhi, Bartonella henselae*, and related zoonotic agents in fleas from domestic cats (*Felis catus*) from the Rio Grande Valley, Texas

**DOI:** 10.1186/s13071-026-07421-1

**Published:** 2026-05-15

**Authors:** Sujata Balasubramanian, Italo B. Zecca, Allyson Koger, Sarah A. Hamer

**Affiliations:** Department of Veterinary Integrative Biosciences, School of Veterinary Medicine, Texas A&M University, Texas, USA

**Keywords:** *Ctenocephalides felis*, *Rickettsia typhi*, Murine typhus, *Bartonella henselae*, Cat scratch disease, Rio Grande Valley, Texas

## Abstract

**Background:**

Flea-borne rickettsiosis and bartonellosis are emerging diseases in humans and animals. There is increasing concern for the emergence of *Rickettsia typhi* (agent of murine typhus or flea-borne typhus) in humans of southern USA, for which cats are key reservoirs and cat fleas (*Ctenocephalides felis*) serve as the vector. Similarly, multiple species of *Bartonella* have cats as natural hosts and fleas as vectors, leading to varied disease outcomes in humans, including cat scratch disease and neurobartonellosis.

**Methods:**

To investigate the ecology of *Rickettsia* and *Bartonella* pathogens, we studied 167 predominantly stray cats (*Felis catus*) from the Rio Grande Valley in south Texas. Fleas were collected, identified morphologically, and confirmed molecularly. DNA from fleas was tested for *Rickettsia* and *Bartonella* using polymerase chain reaction (PCR) and Sanger sequencing of multiple gene targets. Additionally, DNA from blood of all cats and tissues from a subset of cats were assayed for infections.

**Results:**

Flea infestation prevalence of cats was 83.23% (95% CI 76.69–88.56)—higher in male versus female cats—yielding 721 fleas, predominantly *C. felis* with two *Echidnophaga gallinacea* and one *Pulex* sp. Flea burden was a significant predictor of *Rickettsia* infection in fleas. *Candidatus* Rickettsia senegalensis was identified in 28 flea pools (20% of all pools; 95% CI 13.72–27.59) and *Rickettsia typhi* in 6 flea pools (4.29%; 95% CI 1.59–9.09). *Bartonella henselae* was identified in 59 flea pools (42.14% of all pools; 95% CI 33.85–50.77) and blood from 37 cats (22.16%; 95% CI 16.11–29.22); *Bartonella clarridgeiae* was detected in 3 flea pools (2.14% of all pools; 95% CI 0.44–6.13) and blood from 1 cat (0.6%; 95% CI 0.01–3.29). A total of 30 cats had simultaneously *Bartonella-*positive fleas and blood. *Rickettsia* infection was positively associated with flea burden. Coinfections of *Rickettsia* and *Bartonella* in fleas occurred more commonly than expected by chance.

**Conclusions:**

The infection of fleas from cats in south Texas with *R. typhi* and multiple *Bartonella* species, in addition to *Bartonella* infections in the host cats, suggest that public health surveillance for these emergent zoonoses should include a focus on vectors and potential feline reservoirs.

**Supplementary Information:**

The online version contains supplementary material available at 10.1186/s13071-026-07421-1.

## Graphical abstract

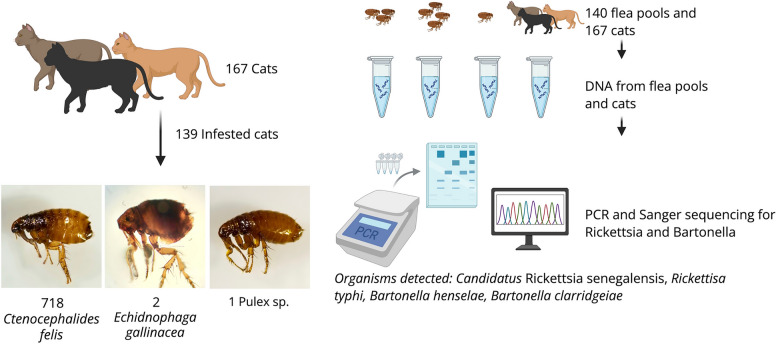

## Background

Flea-borne diseases continue to emerge in human populations worldwide, with increasing attention to pathogens in the genera *Rickettsia* and *Bartonella*. At least 17 species of *Rickettsia* cause disease in humans, many resulting in spotted fever or typhus fever [1, 2]. *Rickettsia typhi*—the causative agent of murine typhus or flea-borne typhus—is an emergent global threat for humans [2–4]. When typhus was initially identified in the 1920s, rats were the reservoirs, and the rat flea (*Xenopsylla cheopis)* was the recognized vector. *Rickettsia typhi* is now also transmitted by *Ctenocephalides felis* (cat flea) and other flea species [5], with opossums (*Didelphis virginiana*) and cats (*Felis catus*) as reservoirs [5, 6]. *Rickettsia typhi* is endemic to south Texas, California, and Hawaii in the USA. Infection with *R. typhi* may cause fever, headache, chills, rash, and gastrointestinal or renal symptoms with potential for hospitalization, though mortality is rare and treatment is accomplished with antibiotics [7]. Clinical impacts on infected pets are not well studied, although infected cats have been reported from many human disease hotspots, and one case study identified *R. typhi* in a clinically ill dog in Texas [8–10].

Multiple species of *Bartonella* are recognized as emerging threats to public health [11]. *Bartonella henselae* is the main causal agent of cat scratch disease but *B. clarridgeiae* is also considered a causal agent [12, 13]. In humans, infection with *Bartonella* has different outcomes for immunocompetent and immunocompromised human hosts [14]. Humans acquire bartonellosis from bites or scratches from cats, more frequently diagnosed in children. Cat-scratch disease in humans progresses acutely from skin lesion to fever and lymphadenopathy. Neurological complications, often ocular, have been observed with *Bartonella* spp. infections [15–17]. In cats, *B. henselae* is maintained in an intra-erythrocytic state and typically results in asymptomatic bacteremia [14, 18], although infected cats may develop cardiovascular issues including lymphoplasmacytic myocarditis among other ailments affecting the lymph nodes, spleen, liver, and kidneys; sometimes fatal [19, 20].

The cat flea (*C. felis*) is a predominant vector for *R. typhi*, *B. henselae*, and related species. This flea has a worldwide distribution and is an abundant and frequent ectoparasite on domestic dogs and cats. Across broad geographic ranges, *C. felis* is the most common flea species found on domestic and feral cats, often among other species including the sticktight flea (*Echidnophaga gallinacea*) and human flea (*Pulex irritans*) [21–25]. In addition to *Rickettsia* and *Bartonella* spp. pathogens, *C. felis* may transmit or carry *Yersinia pestis* (plague), *Dipylidium caninum* (tapeworm), *Hymenolepis diminuta* (rat tapeworm), *Dipetalonema* (*Acanthocheilonema*) *reconditum*, and hemoplasmas [26–28], and may directly impact hosts through flea allergic dermatitis.

Considering the emergent threat of *R. typhi* and *B. henselae* in the USA, our work aims to describe the prevalence of the  genera of these agents among fleas and their cat hosts in the Rio Grande Valley in south Texas.

## Methods

The Rio Grande Valley of south Texas is a region with a subtropical climate that supports many vector species and tropical infections. Cats in our study included 167 predominantly stray cats collected by animal control services from 14 surrounding rural and urban zones of the Rio Grande Valley that were brought to a large shelter in Hidalgo County that intakes an estimated 9000 cats per year; these cats were the subject of a prior study of *Trypanosoma cruzi* and feline Chagas disease [29]. These cats were sampled across winter (1 January–19 March), spring (20 March–20 June), and summer (21 June–19 September) of 2017. At the time of euthanasia for nonstudy reasons, the sex and geographic origin of cats were recorded. Cats were combed using flea combs across the whole body for approximately 3 min and all recovered fleas were preserved in 70% ethanol.

As described previously [29], blood was collected from all cats, and spun and separated in the laboratory with DNA extracted from the blood clot available for the current study. Additionally, DNA extracted from heart was available from all cats and other tissues were also available from cats sampled in spring and summer.

Fleas were identified using the key from Centers for Disease Control and Prevention with pictorial details from Lawrence et al. [30, 31]. When ≤ 5 conspecific fleas were collected from an individual cat, they were pooled for DNA extraction. When > 5 conspecific fleas were present, five were randomly selected to create a pool. When > 1 flea species was present, a pool was made for each species. Representative fleas have been deposited into Texas A&M University Insect Collection (TAMUIC-768).

The Omega EZNA (Omega Bio-tek, Norcross, GA) kit was used to extract DNA from fleas. Fleas were first crushed into lysis buffer using single-use pestles. Negative extraction controls were included in each batch of extraction. Negative extraction controls and a no-template control were included in polymerase chain reaction (PCR) runs, and in all cases, showed no amplification. To confirm the flea species identification, representatives of each species were subjected to a molecular identification process using PCR targeting the insect cytochrome oxidase I locus with primers LCO1490 and HC02198 (Additional file 1: Table S1) [32]. PCR products were sequenced, and chromatograms were analyzed as described below. Representative flea sequences were deposited to Genbank (PP545303-PP545306).

Three different PCRs were used to identify *Rickettsia* (Additional file 1: Table S1) from flea pools and selected cat blood clots and tissues. Outer membrane protein (*rOmpB*) was amplified using primers 120–2788 and 120–3599; this assay can detect a wide range of Rickettsial species [33]. Two sets of primers were used to amplify the *gltA* (citrate synthase) gene: RrCS to detect spotted fever group *Rickettsia* using primers RrCS 372 and RrCS 989 [34] and RpCS to detect the typhus group *Rickettsia* using primers RpCS 877 and 1258n [35]. For the blood and tissue samples from one cat (F159), we also attempted to amplify the *htrA* locus [36].

To detect *Bartonella*, primers of the *pap31* locus of the hemin-binding protein, PAPn1 and PAPn2, were used (Additional file 1: Table S1) [37]; while these primers were initially reported to detect *B. henselae*, we and others and have subsequently used them to amplify additional *Bartonella* species [38, 39]. All PCRs used the Failsafe™ PCR kits (Lucigen, Wisconsin, USA). Resulting PCR fragments were visualized on 1% agarose gel, treated with ExoSapIT (Applied Biosystems, Waltham, MA) and sent to Eton Biosciences (San Diego, CA) for Sanger dideoxy sequencing.

The DNA sequences were analyzed using MEGA7 [40] and Geneious Prime (Biomatters Inc. Boston, MA) and queried against the NCBI Genbank database. We report results of sequences with a minimum match of 97% identity and an E value ≤ 0 to a named species [41]. A sample was called positive only when we retrieved a sequence that met these criteria for at least one locus. Sequences that did not meet these criteria but indicated *Rickettsia* or *Bartonella* were assigned genus-level identification. Sequences have been deposited into GenBank (*gltA*: PP856019-PP856043; *ompB*: PP935775-PP935802; *pap31*: PP943066-PP943101; PP944938-PP944992; PP947812-PP947814).

Flea infestation prevalence of cats was defined as the proportion of sampled cats that harbored at least one flea. Flea burden was defined as the number of fleas per infested host. To test for associations between cat demographic factors (sex, geographic origin) with the outcomes of flea burden and/or flea infection, we used chi-squared and Wilcoxon Rank Sum tests using the program R.

## Results

**Figure 1:**
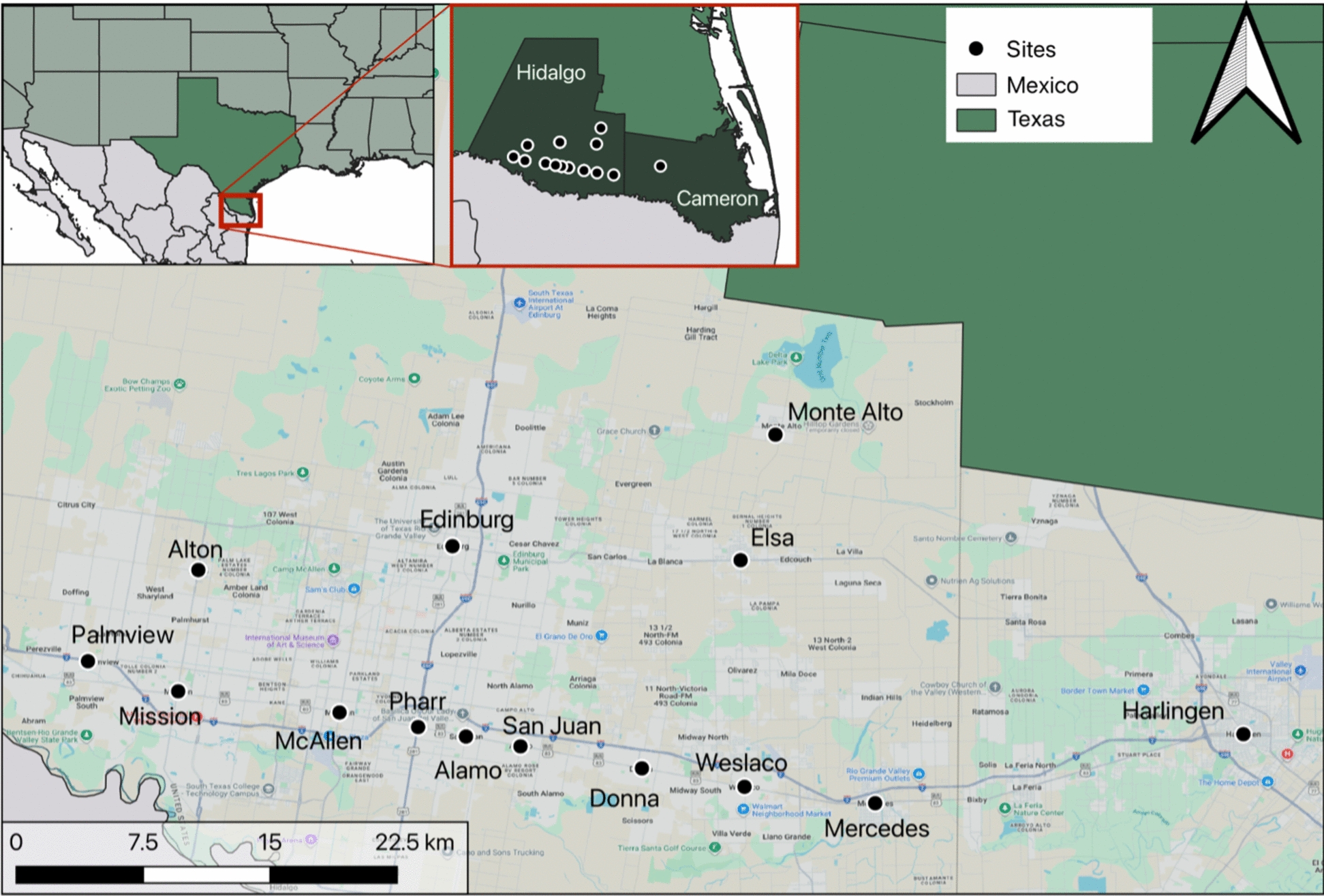
Map showing the locations of origin within Hidalgo County, Texas from where the cats in the study were surrendered. Map was generated using Quantum GIS (QGIS 3.30) using freely available administrative boundaries and map data from OpenStreetMap for satellite imagery. Open Street Map. Planet OSM [Internet]. [cited 2025 Dec 15]. Available from: https://planet.osm.org/

Cats from Hidalgo and Cameron counties in Texas were sampled from January to June 2017. Cats were from 14 different urban locations, with most coming from the cities of McAllen (66), Pharr (28), Edinburg (20), and San Juan (18) (Fig. 1; Table 1). Of the 167 cats in the study, 87 were female and 80 were male. Most cats were sampled in the winter months (72%), with fewer in spring (17%) and summer (11%) (Table 1).

**Table 1:** Infection of fleas removed from cats and cat blood with *Rickettsia* and *Bartonella* across 14 locations of the Rio Grande Valley of south Texas, 2017. Cat blood tested for *Rickettsia* had one positive (F112)

	No. of cats	Flea-infested cats (%)	No. flea pools tested	*Candidatus* Rickettsia senegalensis positive flea pools (%)	*Rickettsia typhi* positive flea pools (%)	*Bartonella henselae* positive flea pools (%)	*Bartonella clarridgeiae* positive flea pools (%)	*Bartonella henselae* positive cat blood (% cats)	*Bartonella clarridgeiae* positive cat blood (% cats)
Location									
Alamo	10	8 (80)	8	3 (38)	1 (13)	6 (75)		5 (50)	
Alton	3	3 (100)	3	2 (67)		2 (67)		1 (33)	
Donna	4	4 (100)	4	1 (25)					
Edinburg	20	14 (70)	14	4 (29)		7 (50)		3 (15)	
Elsa	2	2 (100)	2			2 (100)		1 (50)	
Harlingen	1	1 (100)	1			1 (100)			
McAllen	66	55 (83)	55	10 (18)	4 (7)	16 (29)	1 (2)	5 (8)	
Mercedes	2	1 (50)	1					1 (50)	
Mission	5	5 (100)	5	1 (20)		3 (60)		1 (20)	
Monte Alto	1	1 (100)	1						
Palmview	5	5 (100)	5	1 (20)		2 (40)			
Pharr	28	22 (79)	22	2 (9)	1 (5)	14 (64)	2 (9)	10(36)	
San Juan	18	16 (89)	17	4 (24)		5 (29)		9 (50)	1 (6)
Weslaco	2	2 (100)	2			1 (50)		1 (50)	
Season									
Spring	28	25 (89)	25	1 (4)		8 (32)		10 (36)	
Summer	19	13 (68)	13	2 (15)	1 (8)	6( 46)		1 (5)	
Winter	120	101 (84)	102	25 (25)	5 (5)	45 (44)	3 (3)	26 (22)	1 (1)
Sex									
Female	87	67 (77)	67	8 (12)	3 (4)	32 (48)	1 (1)	19 (22)	1 (1)
Male	80	73 (91)	73	20 (27)	3 (4)	27 (37)	2 (3)	18 (23)	
Total	167	139 (83)	140	28 (20)	6 (4)	59 (42)	3 (2)	37 (22)	1 (1)

A total of 721 fleas were collected from 139 of 167 cats, resulting in an infestation prevalence of 83.23% (95% CI 76.69–88.56) (Table 1). Flea burden ranged from 1 to 41. An average of 4.3 fleas per cat (5.2 fleas per infested cat) were found. Significantly more male than female cats harbored fleas, with 91% and 76% of male and female cats infested, respectively (Wilcoxon rank sum test, W = 2607, *P*-value = 0.004). Cat infestation with fleas occurred across the study, with fleas apparent on 101 of 120 (84%) of cats sampled in winter, 25 of 28 (89%) in spring, and 13 of 19 (68%) in summer. Most fleas (*n* = 718) were *C. felis* (cat flea), with two *Echidnophaga gallinacea* (sticktight flea) and one single *Pulex* sp. Most infested cats harbored only a single flea species, but cat F63 harbored a single *E. gallinacea* and four *C. felis*.

Flea pool size ranged from 1 to 5, with a mean of 3.66 and median of 4 fleas per pool. Of 140 flea pools tested for *Rickettsia,* 34 (24.29%; 95% CI 17.44–32.25) were positive, of which 28 (20% of all flea pools; 95% CI 13.72–27.59) were positive for *Candidatus* Rickettsia senegalensis and 6 (4.29% of all flea pools; 95% CI 1.59–9.09) for *R. typhi*. *Rickettsia*-positive fleas were collected from 9 of the 14 locations, including 14 from McAllen,;4 each from Alamo, Edinburg, and San Juan; 3 from Pharr; 2 from Alton; and 1 each from Donna, Mission, and Palmview. *Rickettsia* infection did not show association with location (chi-squared test, *χ*^2^ = 10.132, df = 13, *P* = 0.683). *Rickettsia* infection was positively associated with flea burden (Wilcoxon rank sum test, W = 968, *P*-value = 1.968 × 10^−07^). A total of 11 of 67 flea pools (16.42%; 95% CI 8.49–27.48) from female cats and 23 of 73 flea pools (31.51%; 95% CI 21.13–43.44) from male cats were positive for *Rickettsia* (chi-squared test, *χ*^2^ = 5.885, df = 1, *P* = 0.0153). Of the six *R. typhi*-positive flea pools, four were from McAllen and one each were from Pharr and Alamo (Table 1).

Additionally, nontarget amplification of *Bartonella* occurred with two *Rickettsia* assays, as one flea pool (F72) amplified using the RrCS *gltA* primers showed a *B. clarridgeiae* sequence; three flea pools amplified using the RpCS *gltA* primers showed *B. henselae* sequences; and five flea pools amplified using the RpCS *gltA* primers resulted in genus-level identifications to *Bartonella*. All the pools that showed *Bartonella* from these two assays were also detected by the *Bartonella*-specific *pap31* PCR (Additional file 1: Table S1).

PCR amplification of the *Bartonella pap 31* locus detected was positive in 90 flea pools (64.29% of all pools; 95% CI 55.75–72.20), of which 59 (42.14% of all pools; 95% CI 33.85–50.77) were confirmed *B. henselae*, 3 (2.14% of all pools; 95% CI 0.44–6.13) *B. clarridgeiae*, and another 28 were identified to the genus level as *Bartonella*. *Bartonella* status was not associated with the location of the host (chi-squared test, *χ*^2^ = 11.135, df = 13, *P* = 0.6). A total of 48 pools from female cats and 42 from male cats carried *Bartonella* (chi-squared test, *χ*^2^ = 0.012238, df = 1, *P*-value = 0.912) (Table 1).

Coinfections of flea pools with both *Rickettsia* and *Bartonella* occurred more commonly than expected by chance (chi-squared test, *χ*2 = 5.858, df = 1, *P*-value = 0.0155), with 25 flea pools positive for both *Rickettsia* and *Bartonella*. This included five of the six *R. typhi*-positive flea pools, which all carried *Bartonella*, of which four were *B. henselae*.

Blood clot and heart tissue DNA from 24 cats was evaluated using the Rickettsial *ompB* locus; these included all 6 cats that harbored *R*. *typhi*-positive fleas, 11 cats that harbored fleas with *Candidatus* Rickettsia senegalensis, and 7 cats with *Rickettsia*-negative fleas. An expanded set of other tissues was evaluated from one cat with *R*. *typhi*-positive fleas (F159) and three with negative fleas (F154, F156, and F157). Although PCR products using *ompB*, *RrCS*, and in one case, *htrA* were observed in some of these samples, only one instance (F112 blood clot) yielded sequence matching *Candidatus* Rickettsia senegalensis. Others did not match *Rickettsia* or yielded poor quality sequences (Additional file 1: Table S2), and thus the cats were considered negative.

Lastly, we examined DNA extracted from blood clots of all 167 cats for *Bartonella* using PCR. A total of 39 samples on the *pap31* PCR were positive—37 (22.16% of cats; 95% CI 16.11–29.22) carried *Bartonella henselae,* 1 (0.6%; 95% CI 0.01–3.29) carried *Bartonella clarridgeiae*, and 1 was only identifiable to genus. When *Bartonella* in cats and fleas were compared, 30 flea pools and matching cats showed carriage of *Bartonella* (chi-squared test, *χ*^2^ = 8.595, df = 1, *P* = 0.0034). The proportion of cats carrying *Bartonella* was highest in cats from San Juan (56%). Seasonally, 28 of 120 (23%) cats sampled in winter showed *Bartonella* (26 *B. henselae*, 1 *B. clarridgeiae*, 1 *Bartonella* spp.), and 10 of 28 (36%) in spring (all *B. henselae*) and 1 of 19 (5%) in summer (1 *B. henselae*). The *B. henselae* infection prevalence was 24% in both female (21/87) and male (19/80) cats.

## Discussion

Our new findings of cat fleas infected with *R. typhi* and multiple *Bartonella* species in south Texas strengthens evidence that cats and their fleas should be considered as potential contributors to the emerging human risk of diseases caused by these agents in south Texas. In a recent study from Galveston, TX [21], *R. typhi* was detected in fleas from 1 of 24 feral cats (4.1%), remarkably similar to 4.3% of 140 flea pools we report here. The Galveston study also detected *Candidatus* Rickettsia senegalensis as we did, in addition to two species that were not apparent in our study, *R. asemboensis* and *R. felis*. In contrast, *R. typhi* was not detected in a survey of fleas from 283 feral/stray cats from southeastern Georgia, USA, in which 16.5% of flea pools were positive for the 17-kDa protein antigen gene of *Rickettsia* spp., none of which were positive for *ompB* of *R. typhi* [42].

Despite the relatively high infection prevalence of *Bartonella* we detected in both vectors (64% of flea pools) and hosts (23% of cats), relatively little ecological work has been done on this agent in the southern USA. In southeastern Georgia, USA, 35.2% of flea pools from feral/stray cats were infected with *Bartonella* [42]. In a study of cats from catteries across North America, a seroprevalence of 35.8% was recorded, in which flea infestation was the most important risk factor for high *B. henselae* seroprevalence [27]. Lower *Bartonella* infection prevalence among fleas removed from cats has been reported in studies from Canada (4.3%), Chile (7.5%), Ethiopia (6%), and Greece (13.5%) [25, 43–45].

As expected, we found that cats with *Bartonella*-positive blood commonly harbored infected fleas, yet there were also several infected flea pools removed from cats whose blood and tissues tested negative, suggesting that those fleas may have picked up the infection from a prior bloodfeeding event on a different host. Similarly, a study of cats in Brazil found *Bartonella* DNA in 47.8% of the cat blood samples and 18.3% of *C. felis* fleas, in which cats infested by positive ectoparasites showed approximately twice the odds of being infected [23].

Two cats (F31 and F33) simultaneously harbored fleas infested with both *R. typhi* and *B*. *henselae*, potentially serving as companion animal sentinels of multiple regional zoonotic threats to humans. Further, this exact sample set of cats has also been examined in previous studies for additional zoonoses, including *Trypanosoma cruzi*, the causal agent of Chagas disease (19/167 cats, 11.4% prevalence), and *Dirofilaria immitis* (canine heartworm), (22/122 cats, 18% prevalence) [29, 46]. In comparing the results from those and this study, two cats (F96 and F141) were infected with *Bartonella*, *T. cruzi*, and *D. immitis*; five (F96, F101, F133, F141, and F142) were infected with *Bartonella* and *T. cruzi*; and eight were infected with *Bartonella* and *D. immitis*. One *T. cruzi*-positive cat harbored *R. typhi*-positive fleas (F35), and two *T. cruzi*-positive cats harbored *Candidatus* Rickettsia senegalensis-positive fleas (F26 and F113). While an older study suggested pet ownership is not a direct risk factor for human exposure to *R. typhi* on the basis of Texas data [47], owning a dog was found to be a risk factor for murine typhus seropositivity in a cross-sectional study from India [48]. While feral/stray cats that remain outdoors may pose little risk of zoonoses transmission from flea importation to the house, those cats that enter homes and those that interact with domestic animals may facilitate “zoonoses in the bedroom” by introducing new disease threats to the domestic setting [49].

Cases of murine typhus have been steadily increasing in the USA over the past three decades, with 348 cases from 1990 to 1999 and 994 from 2000 to 2009 to a stark 3159 from 2010 to 2018 in Texas (https://www.dshs.texas.gov) and 744 cases from California between 2013 and 2019 and 1153 from 2020 to 2025 (https://www.cdph.ca.gov). Within Texas, Hidalgo County, the location of this current study in the Rio Grande Valley of south Texas, appears to be a hotspot for local cases. In the years following this study, 2017–2023, the most cases per county were reported from Hidalgo County for 6 (2017–2020, 2021, 2023) out of 8 years with a total of 710 cases (https://www.dshs.texas.gov). In an 18-year period from 1998 to 2016, 213 pediatric patients were admitted to the hospital in south Texas with a confirmed diagnosis of murine typhus [50].

Nationally, cat scratch disease is diagnosed in  4.5–9.3 patients per 100,000 people, highest in the southern USA, in children aged 5–9 years, and with hospitalization higher among male patients 50–64 years old [12, 51]. In a survey across 9 years of nearly 40 million medical insurance enrollees, 0.03% were diagnosed with cat scratch disease, with nearly one-fifth (19.7%) of cases from the West South–Central USA census region that includes Texas, second to 26.3% in the South Atlantic USA census region [12]. In a similar analysis, over a 10-year period more than half the diagnoses of cat scratch disease were from a southern state in the USA [16]. Although it is established that *Bartonella* infections are life-threatening for immunocompromised individuals, neurobartonellosis in immunocompetent individuals is still incompletely understood [17] and the true public health burden of bartonellosis is unknown. Cats are deemed to be adapted to *B. henselae*, but nonadapted species of *Bartonella* and occasional variants of *B. henselae* can cause cardiac disease in cats [19, 20].

In our study and similar ones, the use of PCR of blood to detect these pathogens has limitations. In humans, *R. typhi* is most readily detected in the first few days after infection or from an eschar sampling. Later stage bacteremia may be below PCR detection limits, and serological tests are thus more useful [52–54]. Further, our use of PCR alone cannot substantiate the roles of *C. felis* as competent vectors of these rickettsial and bartonella agents, nor can it substantiate the role of cats as reservoirs. Finally, we pooled fleas by species when more than one was present on a cat; while this approach allowed for the testing of fleas from all cats at a lower monetary and labor cost, pooling may cause the dilution of signal yielding potential false negatives. New *Rickettsia* and *Bartonella* species and novel vector–host associations continue to be described [55–57], underscoring the need for vector and host surveillance to provide an ecological basis for public and veterinary health risk assessment. Controlling fleas and infections in domestic cats may help to alleviate spillover transmission to humans.

## Conclusions

New *Rickettsia* and *Bartonella* species and novel vector-host associations continue to be described [55–57], underscoring the need for vector and host surveillance to provide an ecological basis for public and veterinary health risk assessment. Controlling fleas and infections in domestic cats may help to alleviate spillover transmission to humans.

## Supplementary Information

**Additional file 1: Table S1.** Primers used in this study are listed alongside the locus and purpose of use. **Table S2.** List of tissues from cats evaluated for Rickettsia with results from PCR listed.

## Data Availability

The datasets generated and/or analyzed during the current study are available in the OAKTrust Digital Repository [https://hdl.handle.net/1969.1/1601334].

## References

[1] Fang R, Blanton LS, Walker DH. Rickettsiae as emerging infectious agents. Clin Lab Med. 2017;37:383–400. 10.1016/j.cll.2017.01.009. 28457356 10.1016/j.cll.2017.01.009

[2] Rodino KG. Rickettsioses in the United States | Elsevier Enhanced Reader. Clin Microbiol Newsl. 2019;41:113–9. 10.1016/j.clinmicnews.2019.06.002.

[3] Azad AF, Radulovic S, Higgins JA, Noden BH, Troyer JM. Flea-borne rickettsioses: ecologic considerations. Emerg Infect Dis. 1997;3:319–27. 9284376 10.3201/eid0303.970308 PMC2627639

[4] Pérez-Osorio CE, Zavala-Velázquez JE, León JJA, Zavala-Castro JE. *Rickettsia felis* as emergent global threat for humans. Emerg Infect Dis. 2008;14:1019–23. 10.3201/eid1407.071656. 18598619 10.3201/eid1407.071656 PMC2600327

[5] Anstead GM. History, rats, fleas, and opossums. II. The decline and resurgence of flea-borne typhus in the United States, 1945–2019. Trop Med Infect Dis. 2020;6:2. 10.3390/tropicalmed6010002. 33379251 10.3390/tropicalmed6010002 PMC7839051

[6] Civen R, Ngo V. Murine typhus: an unrecognized suburban vectorborne disease. Clin Infect Dis. 2008;46:913–8. 10.1086/527443. 18260783 10.1086/527443

[7] Caravedo Martinez MA, Ramírez-Hernández A, Blanton LS. Manifestations and management of flea-borne rickettsioses. Res Rep Trop Med. 2021;12:1–14. 10.2147/RRTM.S274724. 33574726 10.2147/RRTM.S274724 PMC7873028

[8] Juhasz NB, Wilson JM, Haney KN, Clark MH, Davenport AC, Breitschwerdt EB, et al. *Rickettsia typhi* infection in a clinically-ill dog from Houston, Texas. Vet Parasitol Reg Stud Rep. 2022;35:100781. 10.1016/j.vprsr.2022.100781. 10.1016/j.vprsr.2022.100781 36184113

[9] Sorvillo FJ, Gondo B, Emmons R, Ryan P, Waterman SH, Tilzer A, et al. A suburban focus of endemic typhus in Los Angeles county: association with seropositive domestic cats and opossums. Am J Trop Med Hyg. 1993;48:269–73. 10.4269/ajtmh.1993.48.269. 8447530 10.4269/ajtmh.1993.48.269

[10] Gracia MJ, Marcén JM, Pinal R, Calvete C, Rodes D. Prevalence of *Rickettsia* and *Bartonella* species in Spanish cats and their fleas. J Vector Ecol. 2015;40:233–9. 10.1111/jvec.12159. 26611956 10.1111/jvec.12159

[11] Chomel BB, Boulouis H-J, Breitschwerdt EB, Kasten RW, Vayssier-Taussat M, Birtles RJ, et al. Ecological fitness and strategies of adaptation of *Bartonella* species to their hosts and vectors. Vet Res. 2009;40:29. 10.1051/vetres/2009011. 19284965 10.1051/vetres/2009011 PMC2695021

[12] Nelson CA, Saha S, Mead PS. Cat-scratch disease in the United States, 2005–2013. Emerg Infect Dis. 2016;22:1741–6. 10.3201/eid2210.160115. 27648778 10.3201/eid2210.160115 PMC5038427

[13] Kordick DL, Papich MG, Breitschwerdt EB. Efficacy of enrofloxacin or doxycycline for treatment of *Bartonella henselae* or *Bartonella clarridgeiae* infection in cats. Antimicrob Agents Chemother. 1997;41:2448–55. 10.1128/AAC.41.11.2448. 9371348 10.1128/aac.41.11.2448 PMC164143

[14] Jacomo V, Kelly PJ, Raoult D. Natural history of *Bartonella* infections (an exception to Koch’s postulate). Clin Vaccine Immunol. 2002;9:8–18. 10.1128/CDLI.9.1.8-18.2002. 10.1128/CDLI.9.1.8-18.2002 PMC119901 11777823

[15] Jurja S, Stroe AZ, Pundiche MB, Docu Axelerad S, Mateescu G, Micu AO, et al. The clinical profile of cat-scratch disease’s neuro-ophthalmological effects. Brain Sci. 2022;12:217. 10.3390/brainsci12020217. 35203980 10.3390/brainsci12020217 PMC8870711

[16] Nawrocki CC, Max RJ, Marzec NS, Nelson CA. Atypical manifestations of cat-scratch disease, United States, 2005–2014. Emerg Infect Dis. 2020. 10.3201/eid2607.200034. 32568056 10.3201/eid2607.200034 PMC7323523

[17] Delaney S, Robveille C, Maggi RG, Lashnits E, Kingston E, Liedig C, et al. *Bartonella* species bacteremia in association with adult psychosis. Front Psychiatry. 2024;15:1388442. 10.3389/fpsyt.2024.1388442. 38911703 10.3389/fpsyt.2024.1388442 PMC11190357

[18] Rolain JM, La Scola B, Liang Z, Davoust B, Raoult D. Immunofluorescent detection of intraerythrocytic *Bartonella henselae* in naturally infected cats. J Clin Microbiol. 2001;39:2978–80. 10.1128/JCM.39.8.2978-2980.2001. 11474027 10.1128/JCM.39.8.2978-2980.2001 PMC88274

[19] Kordick DL, Brown TT, Shin K, Breitschwerdt EB. Clinical and pathologic evaluation of chronic *Bartonella henselae* or *Bartonella clarridgeiae* infection in cats. J Clin Microbiol. 1999;37:1536–47. 10.1128/JCM.37.5.1536-1547.1999. 10203518 10.1128/jcm.37.5.1536-1547.1999 PMC84823

[20] Breitschwerdt EB, Lappin MR. Feline bartonellosis: we’re just scratching the surface. J Feline Med Surg. 2012;14:609–10. 10.1177/1098612X12458628. 22918842 10.1177/1098612X12458628 PMC10822228

[21] Blanton LS, Vohra RF, Fistein L, Quade B, Walker DH, Bouyer DH. Rickettsiae within the fleas of feral cats in Galveston, Texas. Vector Borne Zoonotic Dis. 2019;19:647–51. 10.1089/vbz.2018.2402. 30835649 10.1089/vbz.2018.2402 PMC6716191

[22] Huang HHH, Power RI, Mathews KO, Ma GC, Bosward KL, Šlapeta J. Cat fleas (*Ctenocephalides felis* clade ‘Sydney’) are dominant fleas on dogs and cats in New South Wales, Australia: presence of flea-borne *Rickettsia felis*, *Bartonella* spp. but absence of *Coxiella burnetii* DNA. Curr Res Parasitol Vector Borne Dis. 2021;1:100045. 10.1016/j.crpvbd.2021.100045. 35284882 10.1016/j.crpvbd.2021.100045 PMC8906117

[23] Raimundo JM, Guimarães A, Amaro GM, da Silva AT, Rodrigues CJBC, Santos HA, et al. Prevalence of *Bartonella* species in shelter cats and their ectoparasites in southeastern Brazil. Rev Bras Parasitol Vet. 2022;31:e014221. 10.1590/s1984-29612022006. 35195184 10.1590/S1984-29612022006 PMC9901869

[24] Razgūnaitė M, Lipatova I, Paulauskas A, Karvelienė B, Riškevičienė V, Radzijevskaja J. *Bartonella* infections in cats and cat fleas in Lithuania. Pathogens. 2021;10:1209. 10.3390/pathogens10091209. 34578241 10.3390/pathogens10091209 PMC8465108

[25] Liodaki M, Spanakos G, Samarkos M, Daikos GL, Christopoulou V, Piperaki E-T. Molecular screening of cat and dog ectoparasites for the presence of *Bartonella* spp. in Attica, Greece. Acta Vet Hung. 2022;70:9–14. 10.1556/004.2022.00004. 35258479 10.1556/004.2022.00004

[26] Bitam I, Dittmar K, Parola P, Whiting MF, Raoult D. Fleas and flea-borne diseases. Int J Infect Dis. 2010;14:e667–76. 10.1016/j.ijid.2009.11.011. 20189862 10.1016/j.ijid.2009.11.011

[27] Foley JE, Chomel B, Kikuchi Y, Yamamoto K, Pedersen NC. Seroprevalence of *Bartonella henselae* in cattery cats: association with cattery hygiene and flea infestation. Vet Q. 1998;20:1–5. 10.1080/01652176.1998.9694824. 9477525 10.1080/01652176.1998.9694824

[28] Iannino F, Sulli N, Maitino A, Pascucci I, Pampiglione G, Salucci S. Fleas of dog and cat: species, biology and flea-borne diseases. Vet Ital. 2017;53:273–5. 10.12834/VetIt.109.303.3. 29307121 10.12834/VetIt.109.303.3

[29] Zecca IB, Hodo CL, Slack S, Auckland L, Rodgers S, Killets KC, et al. Prevalence of *Trypanosoma cruzi* infection and associated histologic findings in domestic cats (*Felis catus*). Vet Parasitol. 2020;278:109014. 10.1016/j.vetpar.2019.109014. 31972512 10.1016/j.vetpar.2019.109014

[30] Lawrence AL, Webb CE, Clark NJ, Halajian A, Mihalca AD, Miret J, et al. Out-of-Africa, human-mediated dispersal of the common cat flea, *Ctenocephalides felis*: the hitchhiker’s guide to world domination. Int J Parasitol. 2019;49:321–36. 10.1016/j.ijpara.2019.01.001. 30858050 10.1016/j.ijpara.2019.01.001

[31] Centers for Disease Control. Pictorial keys to arthropods, reptiles, birds, and mammals of public health significance [Internet]. 1966. https://stacks.cdc.gov. Accessed 15 Dec 2025.

[32] Folmer O, Black M, Hoeh W, Lutz R, Vrijenhoek R. DNA primers for amplification of mitochondrial cytochrome c oxidase subunit I from diverse metazoan invertebrates. Mol Mar Biol Biotechnol. 1994;3:294–9. 7881515

[33] Roux V, Raoult D. Phylogenetic analysis of members of the genus *Rickettsia* using the gene encoding the outer-membrane protein rOmpB (*ompB*). Int J Syst Evol Microbiol. 2000;50:1449–55. 10.1099/00207713-50-4-1449. 10939649 10.1099/00207713-50-4-1449

[34] Williamson PC, Billingsley PM, Teltow GJ, Seals JP, Turnbough MA, Atkinson SF. *Borrelia*, *Ehrlichia*, and *Rickettsia* spp. in ticks removed from persons, Texas, USA. Emerg Infect Dis. 2010;16:441–6. 10.3201/eid1603.091333. 20202419 10.3201/eid1603.091333 PMC3322032

[35] Regenery RL, Spruill CL, Plikaytis BD. Genotypic identification of rickettsiae and estimation of intraspecies sequence divergence for portions of two rickettsial genes. J Bacteriol. 1991;173:1576–89. 10.1128/jb.173.5.1576-1589.1991. 1671856 10.1128/jb.173.5.1576-1589.1991 PMC207306

[36] Webb L, Mitchell C, Malloy DC, Dasch GA, Azad AF. Detection of murine typhus infection in fleas by using the polymerase chain reaction. J Clin Microbiol. 1990;28:530–4. 10.1128/jcm.28.3.530-534.1990. 2108995 10.1128/jcm.28.3.530-534.1990 PMC269657

[37] Zeaiter Z, Fournier P-E, Ogata H, Raoult D. Phylogenetic classification of *Bartonella* species by comparing groEL sequences. Int J Syst Evol Microbiol. 2002;52:165–71. 10.1099/00207713-52-1-165. 11837299 10.1099/00207713-52-1-165

[38] La VD, Tran-Hung L, Aboudharam G, Raoult D, Drancourt M. *Bartonella quintana* in domestic cat. Emerg Infect Dis. 2005;11:1287–9. 10.3201/eid1108.050101. 16102321 10.3201/eid1108.050101 PMC3320506

[39] Tian Y, Juarez JG, Moller-Vasquez AM, Granados-Presa M, Ferreira FC, Pennington PM, et al. Dog ectoparasites as sentinels for pathogenic *Rickettsia* and *Bartonella* in rural Guatemala. Acta Trop. 2024;260:107401. 10.1016/j.actatropica.2024.107401. 39277155 10.1016/j.actatropica.2024.107401 PMC12865852

[40] Kumar S, Stecher G, Tamura K. MEGA7: molecular evolutionary genetics analysis version 7.0 for bigger datasets. Mol Biol Evol. 2016;33:1870–4. 10.1093/molbev/msw054. 27004904 10.1093/molbev/msw054 PMC8210823

[41] Torres-Castro M, Martínez-Ortiz D, Panti-May A, Koyoc-Cardeña E, López-Ávila K, Dzul-Rosado K, et al. *Rickettsia typhi* in rodents from a community with history of murine typhus from Yucatan, Mexico. *Rickettsia typhi* en roedores de una comunidad con antecedentes de tifo murino, de Yucatán, México. Rev MVZ Córdoba. 2018;23:6974–80. 10.21897/rmvz.1420

[42] Brown LD, Maness R, Greer K. Detection of *Bartonella* spp. and *Rickettsia* spp. in cat fleas (*Ctenocephalides felis*) collected from free-roaming domestic cats in southeastern Georgia, USA. Vet Parasitol Reg Stud Rep. 2022. 10.1016/j.vprsr.2022.100743. 10.1016/j.vprsr.2022.100743 35725106

[43] Müller A, Rodríguez E, Walker R, Bittencourt P, Pérez-Macchi S, Gonçalves LR, et al. Occurrence and genetic diversity of *Bartonella* spp. (*Rhizobiales*: *Bartonellaceae*) and *Rickettsia* spp. (*Rickettsiales*: *Rickettsiaceae*) in cat fleas (*Siphonaptera*: *Pulicidae*) from Chile. J Med Entomol. 2018;55:1627–32. 10.1093/jme/tjy124. 30085290 10.1093/jme/tjy124

[44] Kumsa B, Parola P, Raoult D, Socolovschi C. Molecular detection of *Rickettsia felis* and *Bartonella henselae* in dog and cat fleas in Central Oromia, Ethiopia. Am J Trop Med Hyg. 2014;90:457–62. 10.4269/ajtmh.13-0010. 24445204 10.4269/ajtmh.13-0010 PMC3945691

[45] Kamrani A, Parreira VR, Greenwood J, Prescott JF. The prevalence of *Bartonella*, *Hemoplasma*, and *Rickettsia felis* infections in domestic cats and in cat fleas in Ontario. Can J Vet Res. 2008;72:411–9. 19086373 PMC2568045

[46] Mosley IA, Zecca IB, Tyagi N, Harvey TV, Hamer SA, Verocai GG. Occurrence of *Dirofilaria immitis* infection in shelter cats in the Lower Rio Grande Valley region in South Texas, United States, using integrated diagnostic approaches. Vet Parasitol Reg Stud Rep. 2023;41:100871. 10.1016/j.vprsr.2023.100871. 10.1016/j.vprsr.2023.100871 PMC10305843 37208080

[47] Wiggers RJ, Stewart RS. Ownership of cats or dogs does not increase exposure to *Rickettsia typhi*. Tex Med. 2002;98:56–7. 12073903

[48] Devamani CS, Schmidt W-P, Ariyoshi K, Anitha A, Kalaimani S, Prakash JAJ. Risk factors for scrub typhus, murine typhus, and spotted fever seropositivity in urban areas, rural plains, and peri-forest hill villages in South India: a cross-sectional study. Am J Trop Med Hyg. 2020;103:238–48. 10.4269/ajtmh.19-0642. 32458785 10.4269/ajtmh.19-0642 PMC7356468

[49] Chomel BB, Sun B. Zoonoses in the bedroom. Emerg Infect Dis. 2011;17:167–72. 10.3201/eid1702.101070. 21291584 10.3201/eid1702.101070 PMC3298380

[50] Howard A, Fergie J. Murine typhus in South Texas children: an 18-year review. Pediatr Infect Dis J. 2018;37:1071–6. 10.1097/INF.0000000000001954. 29465481 10.1097/INF.0000000000001954

[51] Nelson CA, Moore AR, Perea AE, Mead PS. Cat scratch disease: U.S. clinicians’ experience and knowledge. Zoonoses Public Health. 2018;65:67–73. 10.1111/zph.12368. 28707827 10.1111/zph.12368

[52] CDC. Rickettsial Diseases [Internet]. Yellow Book. 2025. https://www.cdc.gov/yellow-book/hcp/travel-associated-infections-diseases/rickettsial-diseases.html. Accessed 11 Mar 2026.

[53] Rauch J, Eisermann P, Noack B, Mehlhoop U, Muntau B, Schäfer J, et al. Typhus group rickettsiosis, Germany, 2010–2017. Emerg Infect Dis. 2018;24:1213–20. 10.3201/eid2407.180093. 29912688 10.3201/eid2407.180093 PMC6038764

[54] Svraka S, Rolain J-M, Bechah Y, Gatabazi J, Raoult D. *Rickettsia prowazekii* and real-time polymerase chain reaction. Emerg Infect Dis. 2006;12:428–32. 10.3201/eid1203.050888. 16704780 10.3201/eid1203.050888 PMC3291444

[55] do Amaral RB, Cardozo MV, Varani AdeM, Furquim MEC, Dias CM, de Assis WO, et al. First report of *Bartonella* spp. in marsupials from Brazil, with a description of *Bartonella harrusi* sp. nov. and a new proposal for the taxonomic reclassification of species of the genus *Bartonella*. Microorganisms. 2022;10:1609. 10.3390/microorganisms10081609. 36014025 10.3390/microorganisms10081609 PMC9414547

[56] Medkour H, Lo CI, Anani H, Fenollar F, Mediannikov O. *Bartonella massiliensis* sp. nov., a new bacterial species isolated from an *Ornithodoros sonrai* tick from Senegal. New Microbes New Infect. 2019;32:100596. 10.1016/j.nmni.2019.100596. 31719993 10.1016/j.nmni.2019.100596 PMC6839013

[57] Laroche M, Berenger J-M, Mediannikov O, Raoult D, Parola P. Detection of a potential new *Bartonella* species “*Candidatus**Bartonella rondoniensis*” in human biting kissing bugs (*Red**uviidae*; *Triatominae*). PLoS Negl Trop Dis. 2017;11:e0005297. 10.1371/journal.pntd.0005297. 28095503 10.1371/journal.pntd.0005297 PMC5271407

