# Supplementary material for: Rickettsia typhi, Bartonella henselae, and related zoonotic agents in fleas from domestic cats (Felis catus) from the Rio Grande Valley, Texas
Source: Parasit Vectors. 2026 May 15;19:281. doi: 10.1186/s13071-026-07421-1 (PMC13343561; doi:10.1186/s13071-026-07421-1)
Supplement: Supplementary file 1 — Additional file 1: Table S1. Primers used in this study are listed alongside the locus and purpose of use. Table S2. List of tissues from cats evaluated for Rickettsia with results from PCR listed. [file 13071_2026_7421_MOESM1_ESM.docx]

Table S1. Primers used in this study are listed alongside the locus and purpose of use.

| Purpose of PCR | Locus | Primer name and sequence | Reference |
| --- | --- | --- | --- |
| Flea identification | Cytochrome oxidase 1 | LCO1490 5’GGTCAACAAATCATAAAGATATTGG-3’  HC02198 5’TAAACTTCAGGGTGACCAAAAAATCA-3’ | Folmer et al., 1994 |
| *Rickettsia* identification | Outer membrane protein B | 120-2788 5’AAACAATAATCAAGGTACTGT3’  120-3599 5’TACTTCCGGTTACAGCAAAGT3’ | Roux V and Raoult D., 2000 |
|  | Citrate synthase (*Rickettsia rickettsia*) | RrCS 372 5’TTTGTAGCTCTTCTCATCCTATGGC3’  RrCS 989 CCCAAGTTCCTTTAATACTTCTTTGC3’ | Williamson et al, 2010 |
|  | Citrate synthase (*Rickettsia prowazekii*) | RpCS 877 *R. prowazekii* Citrate synthase 5’GGGGGCCTGCTCACGGCGG3’  1258n *R. prowazekii* Citrate synthase ATTGCAAAAAGTACAGTGAACA3’ | Regenery et al., 1991 |
| *Bartonella* identification | Pap31 Hemin binding protein | PAPn1 5’TTCTAGGAGTTGAAACCG3’AT 438-457  PAPn2 GAAACACCACCAGCAACATA3’ 695-714 | Zeaiter et al, 2002 |

Table S2. List of tissues from cats evaluated for *Rickettsia* with results from PCR listed.

| LAB ID | Tissue | *ompB* | *gltA* RrCs |
| --- | --- | --- | --- |
| PV-170104-F23 | Clot | Negative | Negative |
|  | Heart | PCR positive | Negative |
| PV-170203-F26 | Clot | PCR positive | Negative |
|  | Heart | PCR positive | Negative |
| PV-170203-F27 | Clot | PCR positive | Negative |
|  | Heart | PCR positive | Negative |
| PV-170203-F31 | Clot | PCR positive | Negative |
|  | Heart | PCR positive | Negative |
| PV-170203-F33 | Clot | PCR positive | Negative |
|  | Heart | PCR positive | Negative |
| PV-170203-F35 | Clot | Negative | Negative |
|  | Heart | Negative | Negative |
| PV-170203-F38 | Clot | Negative | Negative |
|  | Heart | Negative | Negative |
| PV-170203-F48 | Clot | Negative | Negative |
|  | Heart | Negative | Negative |
| PV-170205-F60 | Clot | Negative | Negative |
|  | Heart | PCR positive | Negative |
| PV-170205-F66 | Clot | PCR positive | Negative |
|  | Heart | Negative | Negative |
| PV-170205-F70 | Clot | Negative | Negative |
|  | Heart | PCR positive | Negative |
| PV-170205-F72 | Clot | Negative | Negative |
|  | Heart | PCR positive | Negative |
| PV-170206-F83 | Clot | PCR positive | Negative |
|  | Heart | PCR positive | Negative |
| PV-170311-F102 | Clot | Negative | Negative |
|  | Heart | PCR positive | Negative |
| PV-170312-F106 | Clot | Negative | Negative |
|  | Heart | Negative | Negative |
| PV-170313-F112 | Clot | *Candidatus* R. senegalensis | Negative |
|  | Heart | Negative | PCR positive |
| PV-170313-F113 | Clot | PCR positive | Negative |
|  | Heart | PCR positive | Negative |
| PV-170313-F115 | Clot | PCR positive | Negative |
|  | Heart | PCR positive | Negative |
| PV-170313-F117 | Clot | PCR positive | Negative |
|  | Heart | Negative | Negative |
| PV-170627-F154 | Clot | PCR positive | Negative |
|  | Heart | PCR positive | Negative |
|  | Colon | PCR positive | Negative |
|  | Distal small intestine | Negative | Negative |
|  | Esophagus | PCR positive | Negative |
|  | Liver | PCR positive | Negative |
|  | Lung | PCR positive | Negative |
|  | Mesentery | PCR positive | Negative |
|  | Middle small intestine | PCR positive | Negative |
|  | Proximal small intestine | PCR positive | Negative |
|  | Stomach | Negative | Negative |
|  | Clot | PCR positive | Negative |
| PV-170627-F156 | Heart | PCR positive | Negative |
|  | Colon | PCR positive | Negative |
|  | Esophagus | PCR positive | Negative |
|  | Mesentery | PCR positive | Negative |
| PV-170627-F157 | Clot | PCR positive | Negative |
|  | Heart | PCR positive | Negative |
|  | Colon | PCR positive | Negative |
|  | Distal small intestine | Negative | Negative |
|  | Esophagus | PCR positive | Negative |
|  | Liver | PCR positive | Negative |
|  | Lung | PCR positive | Negative |
|  | Mesentery | PCR positive | Negative |
|  | Middle small intestine | Negative | Negative |
|  | Proximal small intestine | PCR positive | Negative |
|  | Stomach | PCR positive | Negative |
| PV-170627-F159* | Clot | Negative | Negative |
|  | Heart | Negative | Negative |
|  | Colon | PCR Positive | Negative |
|  | Esophagus | Negative | Negative |
|  | Mesentery | Negative | Negative |
|  | Clot | Negative | Negative |
|  | Heart | Negative | Negative |
|  | Colon | Negative | Negative |
|  | Esophagus | Negative | Negative |
|  | Mesentery | Negative | Negative |

* All the listed tissues from PV-170627-F159, in addition to body fat and sciatic nerve tissue were also assayed using *gltA* RpCS and *htrA* with negative results.
